# Burnout amongst chiropractic faculty, practitioners, and trainees: a scoping review

**DOI:** 10.1186/s12998-024-00550-3

**Published:** 2024-10-07

**Authors:** Brittni L. Partridge, Zachary E. Scott, Christopher B. Roecker, Sheryl A. Walters, Clinton J. Daniels

**Affiliations:** 1 Rehabilitation Care Services, VA Puget Sound Health Care System, Everett, WA 98213 USA; 2 Rehabilitation Care Services, VA Puget Sound Health Care System, Tacoma, WA 98493 USA; 3 Rehabilitation Medicine, University of Washington, Seattle, WA 98195 USA; 4 Logan University, Chesterfield, MO 63017 USA

**Keywords:** Burnout, Professional, Burnout, psychological, Chiropractic, Allied Health personnel

## Abstract

**Objective:**

The purpose of this scoping review was to summarize the literature pertaining to burnout and chiropractic.

**Methods:**

A literature review was performed in accordance with Preferred Reporting of Systematic Reviews and Meta-Analysis Extension for Scoping Reviews (PRISMA-ScR). A literature review was performed by combining the term “chiropractic” with terms relevant to professional burnout (e.g., “work-related stress,” “emotional exhaustion”). We included all publications addressing burnout within the chiropractic profession, including all study designs in only peer-reviewed literature.

**Results:**

Our search yielded 126 citations and 10 met the inclusion criteria. The studies identified consisted of eight surveys and two narrative reviews published from 2011 to 2024. Six of the studies utilized the Maslach Burnout Inventory to assess burnout. Chiropractic students reported greater burnout than the general population. Factors reported to increase burnout risk include higher workload, insurance mandates, and physical demands of daily practice. Factors reported to be protective against burnout included longer duration in clinical practice and philosophy-based practices.

**Conclusions:**

Research on burnout within the chiropractic profession is limited and may not be generalizable. However, the reported factors contributing to burnout are well-documented. Future research should be conducted to improve understanding of the prevalence and causes of burnout in chiropractic.

**Supplementary Information:**

The online version contains supplementary material available at 10.1186/s12998-024-00550-3.

## Introduction

Healthcare professionals are commonly exposed to stressful work environments which “may have a negative impact on mental health, well-being, and lead to burnout” [1–4]. Professional burnout is characterized by “energy depletion, exhaustion, mental distance from one’s job, or feelings of negativism or cynicism” and can lead to a sense of ineffectiveness and lack of accomplishment [4]. Burnout is further characterized by symptoms of emotional exhaustion, depersonalization and a lowered sense of personal accomplishment. While burnout is recognized by the World Health Organization (WHO) [3] and in the International Classification of Diseases (ICD) 11 as an “occupational phenomenon” [5], it is not classified as a distinct mental health disorder by the Diagnostic and Statistical Manual of Mental Disorders (DSM-5) [6].

While burnout is not classified as a mental disorder, this phenomenon may have a substantial impact on healthcare provider’s health and well-being [6]. In May of 2022, the U.S. Surgeon General released an advisory statement titled *Addressing Health Worker Burnout* describing how “burnout manifests in individuals, but it is fundamentally rooted in systems” and called for significant changes to address the “burnout crisis” [7, 8]. The advisory statement also outlined how burnout may worsen staffing shortages, worsen health disparities, negatively impact patients’ clinical outcomes, and disrupt public health response to healthcare-related emergencies [7, 9].

Prior to the COVID-19 pandemic, the National Academy of Medicine reported that burnout had reached “crisis levels” among the U.S. health workforce [10]. With burnout already affecting nearly half of all physicians and medical residents [9, 11], the rates further increased among providers working through the COVID-19 pandemic in high-stress settings (e.g., intensive care units or emergency departments) [12]. American surgeons report a burnout prevalence of 30–50% [13]and prevalence estimates for intensive care unit nurses report high emotional exhaustion (31%), high depersonalization (18%), and low personal accomplishment (46%) [13, 14]. While much has been learned about burnout within other health care professions, little is known about the incidence and prevalence of burnout in the chiropractic profession. However, attempting to gain an understanding of contributing factors to burnout is imperative, and a variety influences have been proposed specific to chiropractors (e.g., isolated and administratively burdensome nature of private practice, negative professional perceptions, risk of work-related injuries, or high student loan default rates) [15]. The purpose of this scoping review was to summarize the state of the available literature, identify gaps, assess the impact of burnout on patient care factors contributing to burnout, and to inform and prompt future research related to burnout in chiropractic professionals and student trainees.

## Methods

This study was performed in accordance with the Preferred Reporting of Systematic Reviews and Meta-Analysis Extension for Scoping Reviews (PRISMA-ScR) [16] and the framework described by Arksey and O’Malley in 2005 [17], which was later updated by Levac et al. in 2010 [18]. The protocol for this scoping review was reviewed by the VA Puget Sound Human Research Protection Office, was determined to be exempt from comprehensive Institutional Review Board (IRB) review, and the protocol was also registered on Open Science Framework [19].

### Stage 1: Identifying the research question

This review addressed the following research questions:

1. *What literature is available that describes workplace or educational burnout for practicing chiropractors or chiropractic trainees?*
2. *What are gaps in the existing literature that warrant additional study?*

### Stage 2: Identifying relevant studies

We searched PubMed, Cumulative Index to Nursing and Allied Health Literature (CINAHL), and the Index to Chiropractic Literature (ICL). Grey literature was searched using PROSPERO, osf.io, clinicaltrials.gov, and the WHO International Clinical Trials Registry Platform (ICTRP). Additionally, we manually searched the references of included papers for relevant citations to identify potentially relevant articles.

The search term “chiropractic” combined with “burnout” and other terms listed in Table 1 were used to identify relevant articles. Peer-reviewed publications were included if they were published between inception of the journal through April 2024.

**Table 1:** Search terms

Search Terms
• Chiropr* AND
• Burnout* • “Burnout” • Burnout, professional • Burnout syndrome • Occupational stress • Chronic work-related stress • Emotional exhaustion disorder • Moral distress • Moral injury • Resilien*

### Stage 3: Study selection

We included peer-reviewed publications utilizing any study design and written in any language. We excluded publications discussing burnout in non-chiropractic health care professions, commentaries, or publications from non-peer reviewed sources (e.g., trade magazines) (Table 2).

**Table 2:** Eligibility criteria

Include	Exclude
• All languages • All study designs • Peer-reviewed articles relevant to burnout and chiropractic professionals or trainees	• Articles relevant to health professionals or trainees that do not include chiropractors • Commentaries or articles from non-peer-reviewed publications (e.g., trade magazines) • Study protocols

**Fig. 1:**
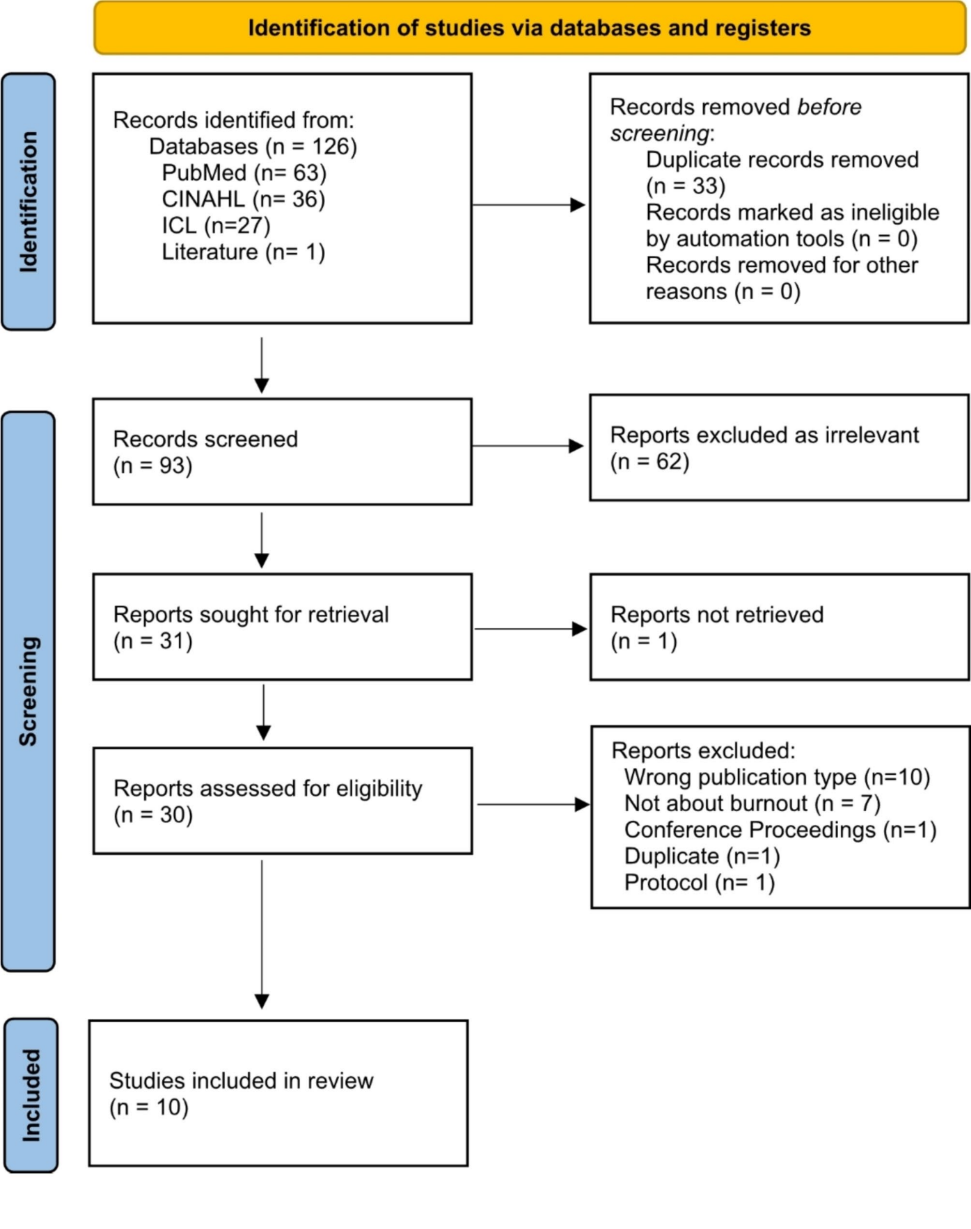
Flow diagram

A health science librarian (S.A.W.) conducted the initial search on July 17, 2023, and then updated the search on April 18, 2024. Citations from the search results were then uploaded to Rayyan, an online evidence synthesis web tool (Qatar Computing Research Institute) [20]. Two co-investigators (B.L.P and Z.E.S.) independently screened all citation titles and abstracts for eligibility, and discrepancies were discussed with a third author (C.B.R.) until a consensus was met. Full text versions of each citation potentially meeting eligibility criteria were acquired and independently screened (B.L.P, Z.E.S., C.B.R.) via the same process. Any citations not meeting the study’s eligibility criteria were discarded, and the reasoning for their exclusion was listed in Fig. 1.

### Stage 4: Charting the data

All articles meeting the eligibility criteria were shared in the Microsoft Teams application (Microsoft Corp., Redmond, WA), and two investigators (B.L.P. and Z.E.S.) independently extracted data from each article. Relevant study data from each article was saved into a Microsoft Excel^®^ spreadsheet. Data items extracted included the lead author’s last name, citation, year of publication, study design, and principal findings. We deviated from the initial study protocol by adding population and outcome measures utilized as additional data items. Formal critical appraisal and pooling of study results was not feasible due to the heterogeneous nature of study participants, burnout screening questionnaires, and study designs.

### Stage 5: Collating, summarizing, and reporting results

A qualitative synthesis of the principal findings of all included citations were organized into potential themes, which were then iteratively reviewed and revised until themes were agreed upon by the investigators.

## Results

The initial search yielded 126 total articles; after duplicates were removed and grey literature was added, 95 articles remained for screening. Screening these 95 individual abstracts yielded 32 potentially relevant articles. The full-text of these 32 articles were evaluated, which resulted in 10 total articles meeting this study’s inclusion criteria. Articles excluded at full text screening are available in Additional File 1. Characteristics of these ten included studies are reported in Table 3.

**Table 3:** Summary of included studies

Author, Citation	Year	Study Design	Population	Outcome Measured	Principle Findings
Tam [23]	2024	Longitudinal Survey	Chiropractic students at Parker University (*n* = 108)	Professional Fulfillment Index (PFI); Maslach Burnout Inventory- Human Services Survey (MBI-HSS)	• Burnout identified through MBI in conjunction with PFI to determine impact on EE • No differences between age or gender • While the percentage of students with burnout increased during statistically significant time points, absolute numbers of students with burnout decreased • Identified as similar rates to other healthcare professions
Ward [28]	2023	Survey	Life Chiropractic College of Chiropractic college faculty (*n* = 43)	Maslach Burnout Inventory (MBI); Epidemic-Pandemic Impacts Inventory (EPII)	• Faculty scored lower on EE and DP subscales and higher on PA subscales compared to other health professional faculties • No significant differences identified between full and part-time faculty • EE was four times higher among faculty who had recently departed the college compared to current faculty
Rigney [30]	2023	Narrative Review	Articles relevant to chiropractic profession attrition	Not applicable	• Burnout listed as a possible cause of attrition within the chiropractic profession • List of causes also included financial burden and questionable business ethics in the profession • Mentions that a provider may continue to stay in the profession regardless of burnout
Etxeberria [21]	2022	Cross sectional and comparative study	Chiropractic students at Barcelona College of Chiropractic (*n* = 69)	Maslach Burnout Inventory-Student Survey (MBI-SS); Perceived Stress Scale (PSS)	• Students in the 2020 COVID-19 lockdown cohort reported lower stress and higher amounts cynicism, compared to the 2018 cohort • Chiropractic students reportedly have higher stress and cynicism compared to the general population • Women reported higher rates of stress and exhaustion, compared to their male peers • Younger students reported higher rates of stress and lower cynicism, compared to their older peer (age 35+)
Alcantara [24]	2021	Cross Sectional Survey	Licensed chiropractors with International Chiropractic Pediatrics Association Membership (*n* = 154)	Safety, Communication, Operational Reliability, and Engagement (SCORE) questionnaire	• Chiropractors reported lower burnout and higher safety rating than the general population • Factors contributing to higher burnout rates included: higher workload, lack of growth opportunities, lack of teamwork climate, poor safety climate, and poor work-life balance • No significant differences were found between genders or age groups
Rank [22]	2021	Cross Sectional Survey	Chiropractic students from 9 European chiropractic institutions (*n* = 121)	Perceived Stress Scale (PSS); Maslach Burnout Inventory (MBI)	• Chiropractic students reported high levels of emotional exhaustion and cynicism, low levels of academic efficacy, and moderate levels of stress • Significant differences in stress and burnout were reported by students at different institutions • No significant differences in burnout were shown based on gender or working status • A positive correlation was reported for rates of cynicism and exhaustion among chiropractic students. • Chiropractic students report higher levels of stress, compared to the general population • Chiropractic students present with burnout scores similar to those of medical, physical therapy, and pharmacy students • Recently accredited schools demonstrated higher levels of burnout and stress compared to older institutions
Williams [27]	2016	Qualitative Survey	Licensed chiropractors identified from a chiropractic marketing agency’s database (*n* = 970)	Non-validated questionnaire involving open-ended questions related to perceived factors related to occupational stressors and emotional exhaustion within the chiropractic profession	• Chiropractors reported the following themes as sources of occupational stress and emotional exhaustion: • Regulations from managed care organizations (MCOs) • Reimbursement from MCOs • Scope of practice issues • Business and administrative • Poor public perception / acceptance
Williams [25]	2014	Cross Sectional Survey	Licensed chiropractors identified from a chiropractic marketing agency’s database (*n* = 1162)	Maslach Burnout Inventory (MBI)	• Burnout among chiropractors was significantly lower than rates reported for medical, nursing, and physical therapy providers. • Factors associated with aspects of burnout include the following: • Dealing with insurance companies • Greater administrative duties • Less time spent providing clinical care • Owning a practice/business • Practice focused on workers’ compensation/personal injury • Musculoskeletal-focused practice • Exposure to opposing chiropractic practice philosophies • Inconsistent public opinion of the chiropractic profession
Williams [26]	2013	Cross Sectional Survey	Chiropractors licensed in New York, New Jersey, and Pennsylvania with contact information in a membership directory (*n* = 90)	Maslach Burnout Inventory-Human Services Survey (MBI-HSS)	• Rates of burnout lower among chiropractors when compared to other professions (i.e., medicine, nursing, physical therapy, occupational therapy, and dentistry) • Factors associated with greater aspects of burnout include the following: • Working in an acute/chronic care setting, compared to a wellness-based setting • History of work-related injury • Poor public perception of the profession • Varying philosophical approach
Williams [29]	2011	Narrative Literature Review	Articles relevant to physical therapy, occupational therapy, dentistry, manual therapy	Not applicable	• Author speculated on potential factors that may contribute to burnout within the chiropractic profession, including: • Physical workload • Role stress Mental and emotional demands

Acronyms: EPII, Epidemic-Pandemic Impacts Inventory; HSS, Health and Human Services Survey; MCO, Managed Care Organizations; MBI, Maslach Burnout Inventory; PFI, Professional Fulfillment Index; PSS, Perceived Stress Scale; SCORE, Safety, Communication, Operational Reliability, and Engagement questionnaire; DP, depersonalization; and EE, emotional exhaustion; PA, personal accomplishment

This scoping review identified three surveys of chiropractic students [21–23], four surveys of licensed chiropractors [24–27], one survey of chiropractic faculty [28], and two narrative reviews [29, 30]. Articles meeting our inclusion criteria were published between 2011 and 2024. Most of the included studies (8/10) were conducted in the United States [23–30], while 2 of the included studies were conducted in Europe [21, 22].

### Burnout outcome measures utilized

Six of the ten articles meeting our inclusion criteria measured burnout using the Maslach Burnout Inventory (MBI), which is considered to be the gold standard questionnaire for identifying burnout [31, 32]. Two studies used the MBI questionnaire, in combination with the Perceived Stress Scale (PSS) [21, 22]. while the remaining two studies used only subsections of the MBI [23, 26]. One study used the SCORE survey (Safety, Communication, Operational Reliability, and Engagement) [24] and two studies generated their own survey instruments in an attempt to target their questions for practicing chiropractors and non-practicing chiropractors that have left the profession [27]. Two studies used additional measures, in combination with the MBI, in order to determine the impact on emotional exhaustion by incorporating the Professional Fulfillment Index (PFI) and the Epidemic-Pandemic Impacts Inventory (EPII) questionnaires [23, 28].

### Licensed chiropractor burnout

Four surveys reported on the experiences of a total of 2,376 licensed chiropractors [24–27]. Many of these studies focused on the rates of burnout within the chiropractic profession, compared to other healthcare professions. Chiropractors surveyed in the Northeastern United States reported lower rates of burnout, when compared against burnout rates for medical providers, nurses, physical therapists, occupational therapists, and dentists [26]. Williams et al. 2013 reported that greater time spent dedicated to administrative duties was associated with statistically increased rates of depersonalization and lower-rated feelings of personal accomplishment. The investigators found that chiropractors working in acute or chronic care settings, experiencing work-related injuries, having diverse philosophical perspectives, and facing negative public perception significantly contributed to burnout. The most common factors reported by surveyed chiropractors as contributing to emotional exhaustion were insurance regulations, limited reimbursement, scope of practice issues, negative public perception, business and administrative duties, and mental and emotional demands [25, 27].

A narrative review proposed a variety of factors potentially contributing to burnout for chiropractors [29]. These included high rates of work-related injury, empathy fatigue, financial concerns secondary to insurance regulation, and market competition with other providers of manual therapy [29]. The study by Williams et al. 2013 reported that chiropractors demonstrated lower rates of burnout when compared to the reported rates of burnout in the medical, nursing, dentistry, and physical therapy professions [26]. A narrative review focusing on attrition from the chiropractic profession suggested burnout as one of many potential causes of attrition and further outlined that chiropractors may still practice while actively experiencing burnout [30].

According to the Maslach Burnout Inventory (MBI), higher levels of emotional exhaustion and depersonalization, as well as lower levels of personal accomplishment are the major factors that lead to professional burnout [32]. The most common factors chiropractors reported as contributing to emotional exhaustion in descending order include insurance regulations (33%), insurance reimbursement (26.8%), scope of practice issues (21.3%), business and administrative duties (16.4%), public perception and acceptance (16.1%), intra-professional stress (13%), and self-perception/purpose (11.2%) [27]. Factors found to be protective against burnout were longer duration of time in practice, philosophy-based approach to practice, older age, being of male gender, and marital status.

### Chiropractic student burnout

Three surveys reported on the experiences of a total of 298 chiropractic students (*n* = 190 European, *n* = 108 US) [21–23]. The two surveys involving European chiropractic students used both the MBI and PSS questionnaires [21, 22]. One of these student surveys identified chiropractic students as having higher rates of burnout, when compared to the general population as well as pharmacy and physical therapy students [22]. Additionally, this survey reported the rates of burnout were higher in more recently accredited institutions (i.e., newer chiropractic programs) [22]. Another survey investigated the impact of the COVID-19 pandemic lockdown on chiropractic students in Spain [21]. Interestingly, this Spanish study reported lower levels of stress during the pandemic when compared to a pre-pandemic cohort of chiropractic students, while heightened levels of cynicism were reported in the COVID-19 cohort. Female chiropractic students involved in this Spanish study reported higher levels of stress and exhaustion when compared to their male counterparts, and younger students reported higher levels of stress as compared to older peers as well as the general population.

The survey involving chiropractic students in the United States followed participants over multiple time points throughout the clinical year of their training and found that approximately half of all students at this stage of their training had burnout [23] and further identified students in clinical term two as having the highest rates of burnout. However, the second term also had the lowest survey response rate. This rate of burnout is consistent with reporting from physicians and other healthcare providers [33, 34].

### Chiropractic college faculty

One survey reported on the experiences of a total of 43 chiropractic faculty members at a single doctor of chiropractic program [28]. Participating faculty scored a lower median for emotional exhaustion and depersonalization subscales, combined with higher levels of personal accomplishment when compared with other healthcare college faculty. Adjunct faculty reported lower emotional exhaustion compared to their full-time peers, and faculty that recently left the college reported emotional exhaustion that was four times higher than the median. Faculty participants who had worked at the college for more than ten years and whose courses did not involve a hands-on component reported higher rates of emotional exhaustion.

## Discussion

Research on burnout within the chiropractic profession remains in its infancy. Our search identified a small number of studies investigating burnout and the majority of the articles identified were cross-sectional surveys involving licensed chiropractors or chiropractic students. There was one longitudinal survey that occurred during the COVID-19 pandemic but this study was limited to 108 students at a single chiropractic institution [23]. Although another study did survey the same student body pre- and post-COVID pandemic, it was not clear if the same individuals participated in each round of this survey (i.e., involved same students in 2018 and 2020) [21]. The other data were obtained through qualitative studies and narrative reviews, offering some insight as to what may cause burnout within the chiropractic profession.

Several themes were identified from the surveys as potential factors for chiropractors developing burnout, many of which were consistent with other healthcare professions. For example, scope of practice issues and inconsistent or negative public opinion regarding the profession may be unique to the field of chiropractic, whereas struggles with insurance mandates, third-party reimbursement, and administrative duties are common sources of burnout across various healthcare disciplines. To date, only one study has attempted to assess burnout within chiropractic program faculty [28]. No studies have attempted to survey burnout symptoms of chiropractors and other health care professionals working in the same type of system (e.g., hospitals or multidisciplinary clinics). It is unclear if the reported burnout symptoms are generalizable across chiropractic professionals or specific to the setting within which they practice. Williams et al. reported that burnout seemed to be higher amongst chiropractors working within acute versus chronic care settings, but it is unclear if this differs from nurses, physicians, or therapists functioning in similar acute and chronic care settings [26].

Burnout is well-documented as an ongoing problem in healthcare, which increases medical errors, malpractice, and possibly even patient mortality [14]. The chiropractic clinical, educational, and administrative repercussions of burnout on patient outcomes and satisfaction remain unknown. Healthcare provider well-being is impacted by burnout and there is an established association with professional attrition, development of alcohol abuse and dependency, and an increased prevalence of suicidal ideation [35]. While it is plausible that repercussions of burnout would have similar a negative impact on chiropractic providers, trainees, and patients, this has yet to be studied.

Currently there are no studies that attempt to address interventions for chiropractic students or providers experiencing burnout. There is evidence that burnout can be successfully addressed through both individual and organizational strategies. However, studies are primarily restricted to single strategies, limited to short-term outcomes, and it is unclear if they are generalizable across healthcare professions [36]. Approaches that individual clinicians may use to combat burnout include stress management training, mindfulness, communication skills training, exercise programs, and other self-care efforts [37–40]. Medical organizations have attempted to prevent and mitigate burnout; approaches that have demonstrated benefit include reducing excessive workloads (e.g., long hours in medical residency and intensive care units), instituting programs to promote efficiency and satisfaction [39] and promoting peer support for healthcare providers [41, 42].

Demographic factors such as age, race, ethnicity, and gender may all play a role in chiropractic profession burnout. Three of the included studies demonstrated no differences in burnout between male and female participants [22–24], whereas one study found that female students had higher levels of stress and exhaustion than their male counterparts [21]. None of the studies investigated burnout symptoms among non-binary chiropractors or student trainees. Similarly, none of the studies compared burnout symptoms among different racial or ethnic groups. The chiropractic profession is mainly Caucasian/white (90.8%) [43] and male (74.6%) and it is unclear if being a majority or minority chiropractic professional is associated with burnout [44].

Future research should strive to incorporate the perspectives and experiences of both current and former chiropractors who may have left the chiropractic profession. Efforts should be made to look at different student and provider subgroups to identify individuals at risk of burnout and to study different interventions for preventing or addressing burnout. We further recommend longitudinal studies that evaluate experiences over multiple time periods to better understand burnout progression, long-term impacts, or methods of mitigation. This may be relevant to chiropractic educational institutions where it is feasible to study burnout over multiple time periods and employ intervention strategies.

The included studies had a wide variety of methodologies, study participant characteristics, burnout definitions, and employed a variety of outcome measures, which complicated our ability to draw overarching conclusions about burnout within the chiropractic profession.

We recommend future studies on burnout within the chiropractic profession utilize the Maslach Burnout Inventory, which is a valid and reliable tool for healthcare professionals, in an attempt to improve consistency and minimize heterogeneity [45]. Consistent use of the MBI may allow for improved analyses and more detailed comparisons across future studies in order to better understand this topic. Studies are also needed to evaluate the impact of chiropractic burnout on patient care, public health, and professional attrition.

### Limitations

There were many limitations to our study. Despite the comprehensive search strategy, a small number of studies were identified, which limits generalized conclusions regarding burnout within the chiropractic profession. Given that the majority of the included studies were surveys of licensed and practicing chiropractors, there may be selection bias that undermines the presence of burnout. It is feasible that the chiropractic profession has experienced attrition due to burnout, and the experiences of these individuals have not been captured in the included surveys. Additionally, there may be response bias present in the surveys, as the chiropractors and students who elected to participate in these studies may have done so because they felt personally impacted by burnout. These potential biases may result in an incomplete understanding of the prevalence and dynamics of burnout in the chiropractic field.

Research on burnout within the chiropractic profession is limited and may not be generalizable beyond the studied populations. The majority of the studies identified by this review were cross-sectional surveys describing individual experiences at a single point in time and are unable to establish the cause of developing burnout symptoms. However, factors that may contribute to burnout are well-documented in healthcare professionals and the results of our study may inform research on this topic and the potential impact of burnout on the chiropractic profession.

## Conclusion

The state of burnout within the chiropractic profession is limited and may not be generalizable. However, the reported factors contributing to burnout are well-documented. Included studies have identified medical errors, malpractice, patient mortality, dependency, and in physicians as impacts on public health and patient care from medical professional burnout. Future research should be conducted to improve understanding of the prevalence and causes of burnout in chiropractic.

## Electronic supplementary material

Below is the link to the electronic supplementary material.

Additional File 1. Excluded citations.

## Data Availability

No datasets were generated or analysed during the current study.

## Abbreviations

DP: Depersonalization
EE: Emotional Exhaustion
PA: Personal accomplishment
HSS: Health and Human Services Survey
MCO: Managed Care Organizations
MBI: Maslach Burnout Inventory
PA: Personal Accomplishment
PSS: Perceived Stress Scale
SCORE: Safety, Communication, Operational Reliability, and Engagement questionnaire
