# Supplementary material for: Burnout amongst chiropractic faculty, practitioners, and trainees: a scoping review
Source: Chiropr Man Therap. 2024 Oct 7;32:29. doi: 10.1186/s12998-024-00550-3 (PMC11459997; doi:10.1186/s12998-024-00550-3)
Supplement: Supplementary file 1 — Additional File 1. Excluded citations. [file 12998_2024_550_MOESM1_ESM.docx]

Additional File 1. Excluded citations.

**Wrong Publication Type**

1. Ferraro, T, Santiago, P. The seven psychological traps every chiropractor must avoid. Chiropractic Journal. 2011;25(5):22.
2. Fueling, T.J. Chiropractic connections - the real risk of stress. Chiropractic Journal. 2001;17(2):26-28.
3. Fueling, T.J. Chiropractic connections - leave your problems at the front door. Chiropractic Journal. 2003;17(12):6.
4. Gentempo, P. The silent dread...chiropractic burnout. Chiropractic Journal. 2001;15(5):37.
5. Kingsigner F, Lawrence D. A proposed bioethics curriculum for chiropractic colleges. Chiropractic Journal of Australia. 2016;44(4).
6. Lloyd N. Cutting staff management stress in half. Chiropractic Journal, p. 4, 2010 Mar.
7. Mertz, C. Have you (lost) what it takes? Chiropractic Journal 2010;18:6.
8. Pallis, K, Plentz E. Burned out, don’t care. Chiropractic Journal. 2004:30.
9. Ressel O. Having fun! Chiropractic Journal. 2010;13:4.
10. Rondberg C. Enthusiasm, information the key to avoid burn out. Chiropractic Journal 1989:21.

**Not about burnout**

1. Hansen MC, Aagaard T, Christensen HW, Hartvigsen J. Work-related acute physical injuries, chronic overuse complaints, and the psychosocial work environment in Danish primary care chiropractic practice - a cross-sectional study. Chiropr Man Therap. 2018 Feb 13;26:4.
2. Innes S, Maurice L, Lastella M, O'Mullan C. Understanding Australian female chiropractors' experiences of inappropriate patient sexual behaviour: a study using Interpretive Phenomenological Analysis. Chiropr Man Therap. 2021 Sep 15;29(1):36.
3. Innes SI. The relationship between levels of resilience and coping styles in chiropractic students and perceived levels of stress and well-being. J Chiropr Educ. 2017;31(1):1-7.
4. Kizhakkeveettil A, Vosko AM, Brash M, Ph D, Philips MA. Perceived stress and fatigue among students in a doctor of chiropractic training program. J Chiropr Educ. 2017 Mar;31(1):8-13.
5. Landman DM, Sewpersadh A, Peterson C. Depression, Anxiety, and Stress Among Chiropractors in South Africa During the Early COVID-19 Pandemic. J Chiropr Humanit. 2022 Oct 1;29:37-43.
6. Patrick K, Lavery J. Sources of stress for chiropractors in private practice. Chiropractor Journal of Australia, vol 36 no. 2. 2006 Jun.
7. Zhang N, Henderson CNR. Coping strategies and chiropractic student perceived stress. J Chiropr Educ. 2022 Mar 1;36(1):13-21.

**Conference Proceeding**

1. Association of Chiropractic Colleges Research Agenda Conference 2020 Abstracts of Proceedings. Platform and poster presentation abstracts [planned to be presented at the 27th ACC-RAC Conference, Dan Diego, California, March 19-21, 2020; cancelled due to the global coronavirus pandemic]

**Duplicate**

1. Williams S, Innes S. Burnout among chiropractic practitioners: real or imagined an exploratory study protocol. Chiropr Man Therap. 2012 Feb 27;20(1):4.

**Protocol**

1. Williams S, Innes S. Burnout among chiropractic practitioners: real or imagined an exploratory study protocol. Chiropr Man Therap. 2012 Feb 27;20(1):4.
